# Sustainable Date Palm Biomass Hydrogel Improves Soil Hydro-Physical Properties and Tomato Growth Under Arid Conditions

**DOI:** 10.3390/gels12020183

**Published:** 2026-02-22

**Authors:** Gamareldawla H. D. Agbna, Syed Javaid Zaidi

**Affiliations:** UNESCO-Chair in Desalination and Water Treatment, Center for Advanced Materials, Qatar University, Doha P.O. Box 2713, Qatar; g.agbna@qu.edu.qa

**Keywords:** agro-waste valorization, arid agriculture, biodegradable hydrogel, date palm biomass, soil water retention

## Abstract

Water scarcity, rapid soil moisture loss, and high evaporative demand severely limit vegetable production in arid regions such as Qatar. Sustainable soil amendments that enhance water retention and stabilize plant water status are therefore critical for improving productivity. This study evaluated a biodegradable hydrogel synthesized from date-palm leaf cellulose using a sodium alginate crosslinking method and assessed its effects on soil hydro-physical properties and tomato (*Solanum lycopersicum* L.) performance under arid conditions. A pot experiment was conducted under semi-controlled conditions using a single-factor randomized complete design with three hydrogel rates (0, 1, and 2% *w*/*w*) and three replications, with one plant per pot. All treatments received the same seasonal irrigation depth, scheduled when soil moisture declined to approximately 60–65% of field capacity. The hydrogel exhibited rapid hydration behavior, reaching equilibrium within 30–60 min with a swelling ratio of 5.659 g g^−1^, corresponding to a water uptake of 465.9%, and SEM analysis revealed a porous internal structure favorable for water retention. At 1 and 2% application rates, hydrogel significantly reduced bulk density, increased total porosity and field capacity, and maintained higher soil moisture across irrigation cycles. Tomato plants grown in hydrogel-amended pots showed substantial gains in fresh biomass and root length, together with higher chlorophyll content, leaf nitrogen concentration, and relative water content. Water use efficiency improved significantly at 1% hydrogel, whereas the 2% rate showed a positive but non-significant trend. Overall, the results demonstrate that hydrogels derived from date-palm waste can enhance soil water retention, plant physiological status, and tomato productivity, offering a locally relevant strategy to improve agricultural resilience in arid environments.

## 1. Introduction

Water scarcity represents one of the most critical challenges facing agricultural production in arid and semi-arid regions, particularly in the Middle East, where climate change and population growth continue to intensify pressure on limited freshwater resources [1]. Qatar has an arid climate with average annual rainfall between 40 and 80 mm and a high natural evaporation rate of approximately 2000 mm per year, which imposes significant limitations on agricultural water management [2]. These environmental conditions necessitate the development and implementation of innovative water-saving technologies to enhance agricultural sustainability and food security in the region [3].

Superabsorbent hydrogels have emerged as promising soil amendments for improving water retention and agricultural productivity in water-limited environments [4,5,6]. These three-dimensional cross-linked polymer networks possess the unique ability to absorb and retain large quantities of water, often 100 to 1500 times their own weight, and release it gradually to plant roots in response to soil moisture depletion [7,8,9]. The incorporation of hydrogels into soil has been shown to reduce irrigation frequency, decrease water consumption, improve soil physical properties, and enhance plant growth and yield across various crops, including vegetables, cereals, and horticultural species [5,10,11].

Commercial agricultural hydrogels predominantly utilize synthetic polyacrylate-based superabsorbent polymers (SAPs), primarily poly(sodium acrylate) and acrylic acid copolymers, which absorb 400–1500 times their own weight in water [7,8,9]. While highly effective, these petroleum-derived SAPs exhibit poor biodegradability (degradation <6% after 500 days), microplastic persistence, and soil structure disruption, raising environmental concerns for long-term agricultural use [4]. In contrast, polysaccharide-based hydrogels from agricultural waste offer biodegradability, renewability, and nutrient compatibility, though typically lower swelling requires optimization for arid conditions [5,6]. Date-palm-derived cellulose–alginate hydrogels address this gap by combining local biomass valorization with proven ionic crosslinking of alginate (Ca^2+^ “egg-box”) and cellulose reinforcement [4,5,6].

It is important to distinguish crosslinked hydrogel systems from conventional organic soil amendments such as compost and biochar, as these categories operate through fundamentally different mechanisms. Compost improves soil primarily through organic matter enrichment, microbial activity stimulation, and gradual nutrient mineralization; its water retention effect is indirect, arising from improved soil aggregation and increased micropore volume, and it lacks the capacity for reversible swelling–deswelling cycles [11,12]. In contrast, crosslinked hydrogels function as engineered three-dimensional polymer networks capable of rapid, reversible water absorption governed by osmotic gradients and ionic crosslink density, absorbing and storing water within their polymer matrix and releasing it gradually in response to soil matric potential changes, a controlled, reversible mechanism entirely absent in composted biomasses [4,5,9]. Published reviews have explicitly classified hydrogels and organic amendments as fundamentally distinct categories of soil water conservation strategies based on their different mechanisms, water retention magnitudes, and temporal effectiveness profiles [11,12,13].

Recent research has demonstrated that hydrogel applications can significantly improve soil water holding capacity, reduce bulk density, and enhance soil porosity, thereby creating a more favorable environment for root development [14]. Studies on various vegetable and cereal crops have documented beneficial effects of hydrogel amendments on plant growth and physiological parameters. In corn cultivation, superabsorbent polymer application enhanced stomatal conductance, transpiration rate, photosynthesis rate, and leaf instantaneous water use efficiency under water deficit conditions [15]. For tomato production, hydrogel soil amendments have been shown to improve growth parameters, increase above-ground and root biomass, enhance leaf area index, and strengthen plant tolerance to drought stress, while also improving water and nutrient use efficiency [16]. However, the efficacy of hydrogels varies considerably depending on their chemical composition, application rate, soil type, and the specific crop being cultivated [12].

In recent years, there has been growing interest in developing biodegradable hydrogels from renewable agricultural waste materials as sustainable alternatives to synthetic petroleum-based polymers that may persist in soil and pose environmental concerns [4,17,18]. Date-palm (*Phoenix dactylifera* L.) residues, which generate approximately 20–35 kg of waste biomass per tree annually and are abundantly available in the Gulf region, with over 28 million date-palm trees in Saudi Arabia alone [19,20], represent a valuable source of lignocellulosic biomass containing 45.3% cellulose [21] that can be converted through carboxymethylation and crosslinking into functional hydrogels with high swelling capacity (400–700% in deionized water) [22], offering dual benefits of waste valorization and production of eco-friendly soil amendments suitable for agricultural applications in arid conditions [23,24,25].

The tomato (*Solanum lycopersicum* L.) is globally significant for its nutritional value but highly sensitive to drought stress, which significantly impairs yield and fruit quality [26,27,28,29]. In Qatar, tomatoes are central to its food security strategy, with production nearly doubling from 14,563 tons (2017) to 26,133 tons (2018), contributing to the goal of 70% self-sufficiency in greenhouse vegetables by 2030 [30].

Superabsorbent hydrogels have been widely investigated as soil amendments to enhance soil water retention and alleviate drought stress in agricultural systems [5,9,11]. Previous studies have demonstrated that hydrogel application can increase soil water-holding capacity, reduce moisture loss, and improve crop growth and water use efficiency under limited irrigation conditions [9,12,16]. However, the agronomic effectiveness of hydrogel amendments varies considerably depending on polymer composition, crosslinking structure, soil texture, crop species, and irrigation regime, and many studies evaluate either material properties or plant responses in isolation [5,11,12]. Moreover, systematic investigations that simultaneously couple detailed soil hydro-physical characterization with crop morphological and physiological responses under extreme arid greenhouse conditions remain limited, particularly for biodegradable hydrogels derived from locally available agricultural residues. To date, no published work has evaluated a cellulose–alginate hydrogel produced specifically from local date-palm leaf residues for vegetable production in Qatar while simultaneously quantifying soil water retention, tomato growth, and water use efficiency under a common irrigation regime. Addressing this gap is essential to determine whether locally sourced hydrogels can provide a technically effective and environmentally compatible tool to enhance the resilience of protected agriculture in arid coastal environments. This study explores the use of biodegradable hydrogel synthesized from locally sourced date-palm leaves to enhance tomato (*Solanum lycopersicum* L.) cultivation under Gulf conditions. It hypothesizes that hydrogel application will improve soil water retention capacity and bulk density while enhancing plant morphological development, physiological status, and overall growth performance. To test this hypothesis, the study aimed to: (i) synthesize a biodegradable hydrogel from date-palm leaves, (ii) evaluate its effects on soil water retention and bulk density, and (iii) assess its impact on tomato morphological, physiological, and biomass parameters.

## 2. Results and Discussion

### 2.1. Structural and Chemical Characterization of the Date-Palm Hydrogel

**Figure 1:**
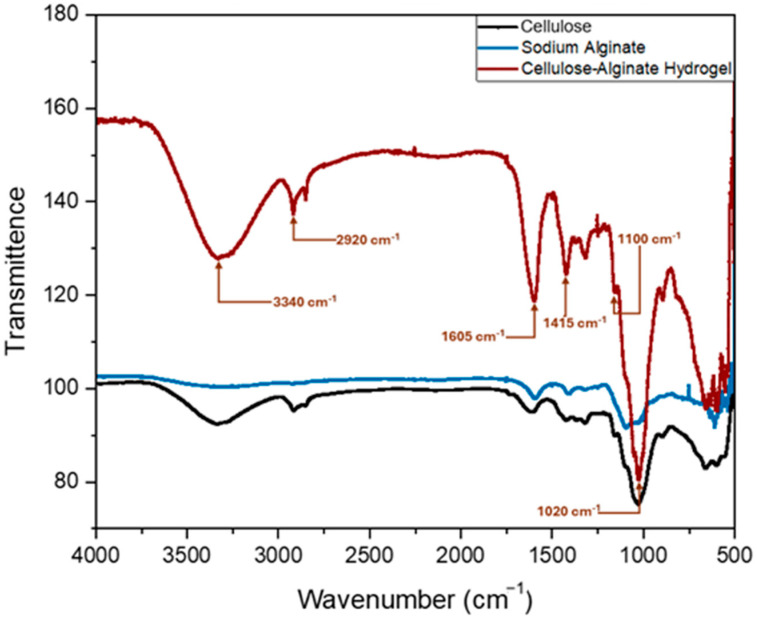
FTIR spectrum of the dried date-palm cellulose–alginate composite hydrogel. The spectrum confirms the presence of both alginate and cellulose components in the hydrogel, with the following characteristic polysaccharide bands: broad O–H stretching at 3340 cm^−1^ (hydrogen bonding in cellulose and alginate); aliphatic C–H stretching at 2920 cm^−1^; asymmetric COO^−^ stretching at 1605 cm^−1^ (diagnostic of alginate and indicative of Ca^2+^–alginate ionic crosslinking); and C–O–C glycosidic linkage/C–O stretching at 1020–1100 cm^−1^ (confirming preserved polysaccharide structure of both cellulose and alginate).

FT-IR analysis was performed on dried hydrogel samples using a Nicolet 760 FT-IR spectrometer (Figure 1) and confirmed the presence of characteristic polysaccharide functional groups, with clear and distinct peak assignments across the spectrum. A broad, intense absorption band centered at 3340 cm^−1^ is characteristic of O–H stretching vibrations in cellulose and alginate, reflecting extensive hydrogen bonding between hydroxyl groups and water molecules that underpins the hydrogel’s hydrophilic nature and high water absorption capacity. A distinct peak at 2920 cm^−1^ corresponds to asymmetric C–H stretching vibrations typical of the aliphatic backbone of both polymers. A sharp, well-defined band at 1605 cm^−1^ is assigned to the asymmetric stretching vibration of carboxylate groups (COO^−^), characteristic of alginate and confirming successful incorporation of the alginate crosslinking component; this band is diagnostic of calcium–alginate ionic interactions (Ca^2+^–COO^−^) that stabilize the three-dimensional hydrogel network. A pronounced peak at 1415 cm^−1^ corresponds to O–H in-plane bending vibrations of primary alcohol groups on the cellulose backbone, indicating preservation of cellulose structural integrity during synthesis.

In the fingerprint region, strong, closely spaced peaks between 1020 and 1100 cm^−1^, with pronounced maxima at 1070 and 1035 cm^−1^, correspond to C–O–C glycosidic linkages and C–O stretching vibrations characteristic of polysaccharide-based polymers. These peaks confirm the presence of cellulose and alginate components in the composite hydrogel and are consistent with spectral features reported for cellulose-based and alginate-containing hydrogels. These spectral features are consistent with FT-IR patterns reported for date-palm-derived cellulose and biomass-based hydrogels [22,31].

**Figure 2:**
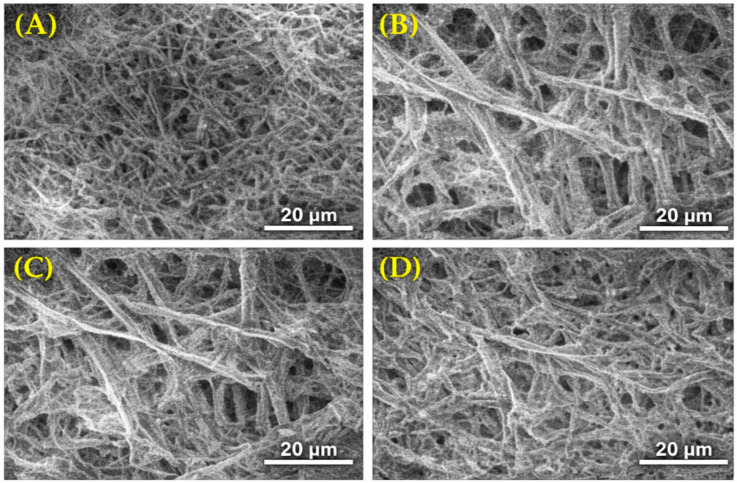
Multi-scale SEM micrographs of freeze-dried date-palm cellulose–alginate composite hydrogel. (**A**) Low-magnification view showing the rough, irregular surface morphology characteristic of a composite material. (**B**–**D**) Higher-magnification internal cross-sections revealing a heterogeneous, interconnected porous network with cellulose microfibrils physically embedded within the continuous alginate gel matrix.

SEM micrographs (Figure 2) reveal a heterogeneous, porous three-dimensional architecture characterized by an interconnected network of fibrillar structures and voids distributed across multiple length scales. Importantly, the observed morphology is characteristic of a cellulose-reinforced alginate hydrogel and is distinct from the relatively uniform fibrillar networks typically reported for pure cellulose substrates [32,33]. At a lower magnification (Figure 2A), the hydrogel exhibits a rough and irregular surface morphology with continuous gel-phase domains interspersed with fibrillar elements, reflecting the composite nature of the material, while higher-magnification images (Figure 2B–D) show internal cross-sections composed of intertwined cellulose microfibrils embedded within the alginate matrix.

The composite architecture of cellulose microfibrils being physically embedded within a continuous alginate gel matrix, rather than forming a self-supporting cellulosic network, is the defining morphological feature of cellulose-reinforced alginate hydrogels [32,33]. The visible cellulose fibrils confirm successful physical incorporation of date-palm-derived cellulose within the alginate network, providing fibrillar reinforcement that contributes to the structural complexity and multi-scale porosity of the hydrogel.

The resulting hierarchical pore structure, consisting of interconnected micro- and meso-scale channels, facilitates water accommodation and diffusion within the polymer matrix and enhances physical interaction and mechanical interlocking with surrounding soil particles when applied as a soil amendment. This microstructural organization is consistent with the improved soil water retention and plant growth responses observed in subsequent agronomic evaluations.

### 2.2. Swelling Behavior, Water Uptake, and Implications for Soil Water Retention

**Figure 3:**
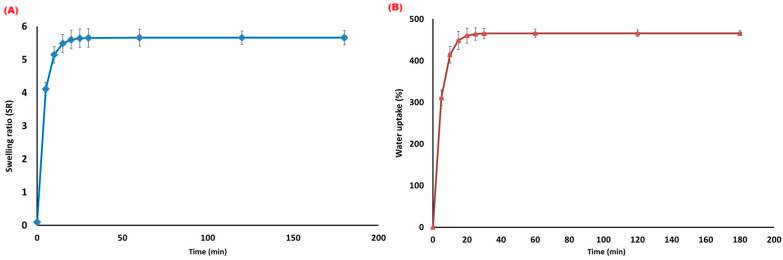
Swelling behavior of the date-palm-derived hydrogel as a function of time, expressed as (**A**) swelling ratio (SR = W_t_/W_d) and (**B**) water uptake (%), calculated as ((Wₜ − W_d)/W_d) × 100. The results show rapid initial hydration followed by equilibrium swelling, with water uptake trends corresponding directly to the swelling ratio.

The hydrogel demonstrated rapid water absorption during early immersion, exhibiting a characteristic biphasic kinetic profile (Figure 3A,B). The swelling ratio (SR), defined as grams of water absorbed per gram of dry hydrogel (g g^−1^), increased from 0.10 g g^−1^ at t = 0 min to 4.108 g g^−1^ within the first 5 min (4100% increase over dry mass), reaching equilibrium at 5.659 g g^−1^ by 30 min and remaining stable through 180 min (Figure 3A). Correspondingly, water uptake (%), calculated as percentage mass increase relative to initial dry weight, rose from 0.10% at 0 min to 310.8% at 5 min, reaching equilibrium at 465.9% by 30 min (Figure 3B). These two metrics are mathematically related: the SR of 5.659 g g^−1^ yields water uptake = (5.659 − 1) × 100 = 465.9%, confirming complete consistency between measurements.

The absorption profile exhibits two distinct kinetic phases: (1) a rapid initial phase (0–5 min) corresponding to surface and macropore filling, and (2) a slower equilibration phase (5–30 min) reflecting osmotic penetration into the polymeric network. This pattern is characteristic of natural polysaccharide hydrogels. The equilibrium SR (~5.66 g g^−1^) and water uptake (~466%) fall within the range previously reported for biomass-derived hydrogels, including date-palm waste (400–700% in deionized water) [22,25,31], confirming material-typical performance suitable for soil moisture buffering under arid conditions.

These hydration characteristics directly explain the improved soil water retention observed in hydrogel-treated pots. By absorbing and storing part of the irrigation water, the hydrogel acts as a reservoir that slowly releases moisture as soil dries, thereby increasing available water and stabilizing moisture dynamics under arid growing conditions [4,9,11]. The porous morphology observed by SEM is consistent with previous reports on alginate–cellulose composite hydrogels [16,25] and increases the surface area for water absorption, facilitates rapid imbibition, and provides diffusion pathways for water release back into the soil—all essential for moderating moisture fluctuations under arid conditions.

### 2.3. Effects on Soil Hydro-Physical Properties

Hydrogel application significantly modified key hydro-physical parameters of the tomato growth medium (Table 1). Bulk density decreased from 1.38 g cm^−3^ in the control (T1) to 1.27 g cm^−3^ in the 1% treatment (T2) and 1.22 g cm^−3^ in the 2% treatment (T3), with both hydrogel treatments significantly different from T1 (*p* < 0.05). Correspondingly, total porosity increased from 47.92% in T1 to 51.82% and 54.10% in T2 and T3, respectively, with significant differences relative to the control (*p* < 0.05).

These changes confirm the hydrogel’s role in improving the soil’s physical structure. Hydrogels act as low-density, expandable inclusions that disrupt dense packing and generate additional pore space upon swelling, thereby enhancing root penetration and gas exchange. Similar reductions in bulk density and increases in porosity have been reported for sandy or coarse-textured soils amended with superabsorbent polymers [10,14].

Field capacity rose from 21.17% in T1 to 30.16% and 30.93% in T2 and T3, with both hydrogel treatments significantly higher than the control (*p* < 0.05). The wilting point increased moderately with hydrogel addition (from 8% to 10.2% in T2 and 11.1% in T3, *p* < 0.01), but the available water (FC − WP) still increased substantially from 13.13% in T1 to approximately 20% in T2 and T3 (*p* < 0.05). The plant-available water content (PAWC) improved from 0.18 g g^−1^ in T1 to 0.25 g g^−1^ in T2 (*p* < 0.05), with T3 slightly lower (0.24 g g^−1^) and not significantly different from the control (ns).

This pattern aligns with the concept that hydrogels expand the storage domain for plant-accessible water without proportionally increasing the water held at tensions beyond the root extraction threshold [6,12]. The pronounced increase in field capacity, coupled with a smaller rise in wilting point, resulted in substantial gains in available water, consistent with observations that hydrogel amendments can increase soil water holding capacity by 20–80% in arid and semi-arid soils [11,15].

**Figure 4:**
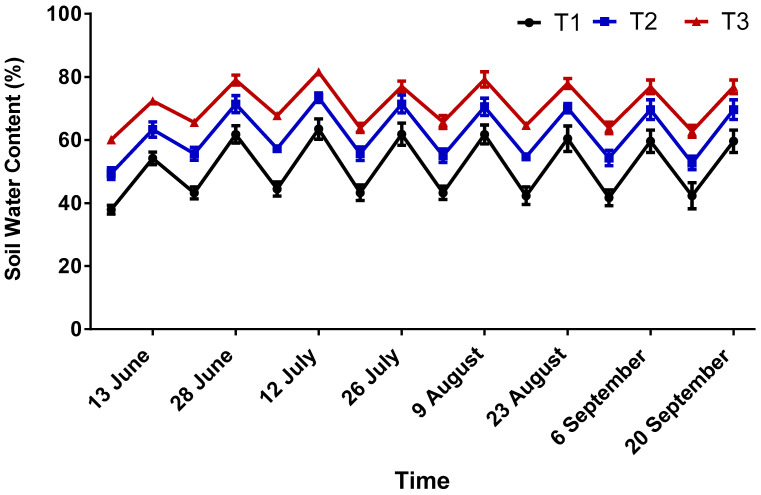
Soil water content (%) in tomato cultivation under different date-palm hydrogel application rates (T1 = 0%, T2 = 1%, T3 = 2% *w*/*w*) throughout the growing season. Values represent means of three replicates.

Soil water content exhibited clear differences among treatments throughout the growing period (Figure 4). In general, pots amended with hydrogels (T2 and T3) maintained a higher soil moisture than the control, especially during drying intervals between irrigation events. This pattern was more evident from the mid-season onwards, when atmospheric demand increased and water depletion accelerated. The higher soil water content recorded in hydrogel treatments confirms the hydrogels’ ability to stabilize moisture dynamics under high evaporative demand. By storing a portion of irrigation water within their polymer matrix, hydrogels slow down soil drying and maintain more favorable water status between irrigation events. Similar behavior has been documented in maize, sorghum, and vegetable systems, where hydrogels extended irrigation intervals and reduced water stress [10,15]. In the present experiment, the advantage became increasingly visible during late-season periods when temperatures and transpiration demand were highest, which is particularly relevant for Qatar’s climate, where high evaporative demand is a major constraint on protected agriculture [2,10,26,27].

### 2.4. Plant Growth and Morphological Responses

**Figure 5:**
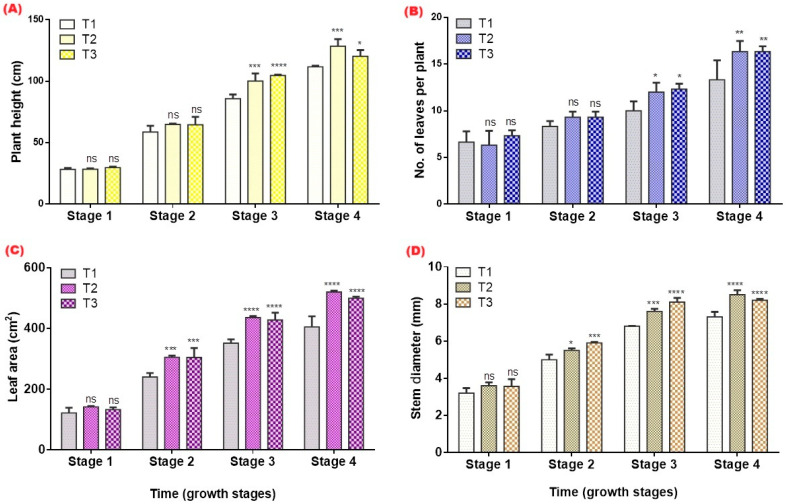
Plant height (cm) (**A**), number of leaves per plant (**B**), leaf area (cm^2^) (**C**), and stem diameter (mm) (**D**) of tomato plants grown under different hydrogel application rates (T1 = 0%, T2 = 1%, T3 = 2% *w*/*w*) during the cropping season. Values represent means of three replicates. Values marked with * are significantly different from T1 (control) at *p* < 0.05 according to Tukey’s multiple comparison test. Significance levels: ns = not significant; * = *p* ≤ 0.05; ** = *p* ≤ 0.01; *** = *p* ≤ 0.001; **** = *p* ≤ 0.0001 for hydrogel treatments.

Tomato plants grown with hydrogel exhibited greater height compared with the control throughout the growing season (Figure 5). Both the T2 (1%) and T3 (2%) treatments showed continuous increases in plant height (Figure 5A), with significant differences from T1 at multiple measurement points (*p* ≤ 0.05). The height advantage became more pronounced after the mid-vegetative stage, indicating that improved soil moisture conditions supported sustained vertical growth under the same irrigation schedule.

Hydrogel application also significantly increased the number of leaves per plant (Figure 5B). Leaf production in T2 and T3 was consistently higher than in T1, particularly during the active vegetative phase, with several measurement dates showing significant differences. This enhanced leaf proliferation reflects improved plant vigor and water availability in the root zone. Leaf area increased markedly in plants receiving hydrogel amendments (Figure 5C). Both T2 and T3 exhibited larger leaf areas than the control across the season, with significant differences at several observation points. The expansion of leaf area suggests enhanced cell expansion and turgor maintenance due to improved soil moisture retention in hydrogel-amended pots.

Stem diameter was greater in T2 and T3 compared with the control throughout the experiment, with significant differences recorded on several dates (Figure 5D). The increased stem girth indicates that improved structural strength and better water supply support vegetative growth.

These improvements in plant height, leaf number, leaf area, and stem diameter indicate that tomato plants benefited from a more favorable root-zone environment. Enhanced vegetative growth under hydrogel amendments has been reported for tomatoes and other horticultural crops, often attributed to increased soil moisture and better root development [5,16]. The higher leaf areas and stronger stems suggest that plants were able to sustain cell expansion and maintain turgor for longer during drying cycles, reducing growth interruptions caused by short-term water deficits.

### 2.5. Physiological Status and Nutrient Relations

**Figure 6:**
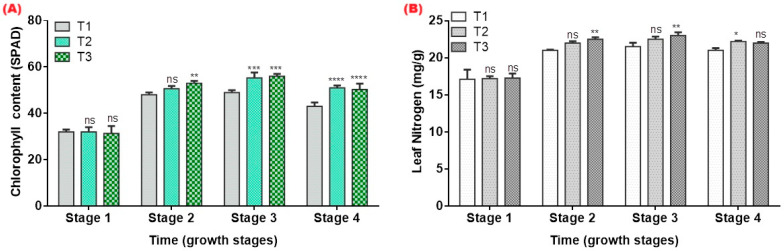
Chlorophyll content (SPAD units) (**A**) and leaf nitrogen concentration (%) (**B**) in tomato leaves under different hydrogel application rates (T1 = 0%, T2 = 1%, T3 = 2% *w*/*w*) during the cropping season. Values represent means of three replicates. Values marked with * are significantly different from T1 (control) at *p* < 0.05 according to Tukey’s multiple comparison test. Significance levels: ns = not significant; * = *p* ≤ 0.05; ** = *p* ≤ 0.01; *** = *p* ≤ 0.001; **** = *p* ≤ 0.0001 for hydrogel treatments.

Chlorophyll content increased in tomato plants receiving hydrogel treatments compared with the control (Figure 6). Both T2 and T3 consistently recorded higher SPAD values throughout the season, with significant differences from T1 at multiple measurement dates (*p* ≤ 0.05) (Figure 6A). The enhanced chlorophyll concentration indicates improved leaf physiological status and better maintenance of photosynthetic capacity under the same irrigation regime. These findings agree with reports that hydrogel-amended soils often support improved leaf greenness and photosynthetic capacity, particularly under deficient irrigation [15,16].

Hydrogel-amended plants exhibited higher leaf nitrogen concentrations than the control across most sampling points (Figure 6B). Both T2 and T3 showed significant increases in N content at several dates, reflecting enhanced nitrogen availability and uptake. The improved nutrient status corresponds with higher soil moisture retention in hydrogel treatments, which reduces nutrient loss and supports more efficient nutrient assimilation. Hydrogels can reduce nutrient leaching and enhance the residence time of soluble fertilizers in the root zone, thereby increasing nutrient use efficiency [9,16].

### 2.6. Biomass Accumulation

Hydrogel treatments significantly improved biomass production (Table 2). Plant fresh weight (PFW) increased from 247.68 g in T1 to 341.70 g in T2 (38% increase) and 331.80 g in T3 (34% increase), both significantly higher than the control (*p* < 0.01). Plant dry weight (PDW) rose from 49.00 g in T1 to 68.68 g in T2, with T2 significantly higher than the control (*p* < 0.05), while T3 showed a similar mean (68.00 g) but was not statistically different from T1. This pattern demonstrates that while hydrogel application consistently enhanced fresh biomass accumulation across both application rates, the improvement in dry matter production was significant only at the 1% rate, indicating variable effects across treatments.

Root fresh weight increased from 36 g in the control to 42 g in T2 and 47 g in T3, with the highest level of significance observed in T3 (*p* < 0.0001). Root dry weight followed the same trend, rising from 3.57 g in T1 to 4.48 g in T2 and 4.60 g in T3, respectively. Root length increased from 19.6 cm in the control to 25.1 cm in T2 and 25.7 cm in T3, with both hydrogel treatments significantly higher than T1. The increases in root mass and length are especially important in sandy substrates, where enhanced root exploration can improve access to both stored soil moisture and water released from the hydrogel matrix.

Hydrogel application clearly improved root biomass, particularly for the 2% treatment. However, the pattern for above-ground biomass was more nuanced: fresh weight improved significantly at both rates, but dry matter production showed significant gains only at the 1% rate. Similar biomass gains have been reported in tomatoes, maize, and other crops grown in hydrogel-amended soils under water-limited conditions [10,14,16].

### 2.7. Leaf Relative Water Content and Water Use Efficiency

**Figure 7:**
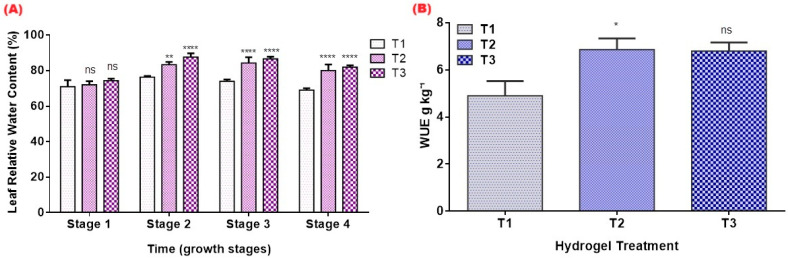
Leaf relative water content (LRWC, %) (**A**) and water use efficiency (g kg^−1^) (WUE) (**B**) in tomato plants cultivated under different hydrogel application rates (T1 = 0%, T2 = 1%, T3 = 2% *w*/*w*) during the cropping season. Values represent means of three replicates. Values marked with * are significantly different from T1 (control) at *p* < 0.05 according to Tukey’s multiple comparison test. Significance levels: ns = not significant; * = *p* ≤ 0.05; ** = *p* ≤ 0.01; **** = *p* ≤ 0.0001 for hydrogel treatments.

Leaf relative water content was markedly higher in hydrogel treatments compared with T1 (Figure 7A). The difference was most pronounced during mid- to late-season measurements, when evapotranspiration demand increased. Both T2 and T3 maintained significantly higher LRWC values at several measurement dates, demonstrating improved leaf hydration and delayed onset of water stress despite identical irrigation volumes. Elevated leaf relative water content in T2 and T3 indicates that plants maintained better tissue hydration under the same irrigation schedule. This is consistent with previous studies showing that hydrogels help maintain leaf water status and delay the onset of physiological drought symptoms, thereby supporting higher stomatal conductance and photosynthesis during dry periods [5,15].

Water use efficiency improved under hydrogel application, but with important treatment-dependent variations (Figure 7B). WUE in the 1% treatment (T2) was significantly higher than the control (*p* < 0.05), indicating that moderate hydrogel incorporation enabled plants to convert a fixed irrigation volume into greater biomass. In contrast, the 2% treatment (T3) produced a higher mean biomass but did not significantly outperform the control in WUE, suggesting that further increases in the hydrogel rate did not translate into proportional gains in water productivity under the present irrigation schedule and pot constraints. Similar non-linear responses have been reported in other short-duration hydrogel studies, where improved growth was not always accompanied by uniformly higher WUE because of limited water stress duration and the absence of graded deficit irrigation treatments [11,34]. These findings point to 1% *w*/*w* as a potentially efficient rate for this soil–crop system and underline the need for longer-term field experiments with explicit water-saving scenarios to fully quantify hydrogels’ contribution to irrigation reduction in arid agriculture.

### 2.8. Comparative Advantages of Cellulose-Reinforced Hydrogels

Polysaccharide-based hydrogels such as cellulose and alginate composites are recognized as biodegradable, biocompatible alternatives to petroleum-derived superabsorbent polymers for agricultural water management [13]. Natural polymer hydrogels improve soil moisture retention and plant biomass under drought stress, though with lower maximum swelling than conventional synthetic polymers [13,35,36,37]. Cellulose incorporation into alginate matrices enhances mechanical integrity and slows structural degradation during repeated wetting–drying cycles compared to pure alginate matrices, thereby extending functional longevity in soil environments [32,33]. The improved plant biomass (+38% fresh weight) and water use efficiency (+17%) at the 1% hydrogel rate in this study align with documented benefits of polysaccharide-based hydrogels in arid agricultural systems [37].

The date-palm cellulose-alginate composite hydrogels used in this study exemplify the synergistic advantages of combining cellulose with alginate matrices. These cellulose-reinforced alginate composites applied as soil conditioners improve soil structure, moisture retention, and nutrient availability while reducing nutrient leaching [35]. Cellulose–alginate hydrogels offer enhanced water retention mechanisms and significant environmental advantages over acrylic superabsorbent polymers (SAPs), which persist in soil for extended periods and pose long-term ecological concerns [33]. This specific combination of cellulose and alginate provides a sustainable balance between water retention capacity and environmental compatibility, supporting their potential as soil amendments under water-limited conditions and water-scarce regions [13,33,37].

The role of cellulose as a structural reinforcing agent within polysaccharide hydrogel matrices is well documented. Cellulose microfibrils enhance the mechanical integrity of hydrogel networks through hydrogen bonding and physical entanglement, contributing to improved structural stability during repeated wetting–drying cycles [32,33]. In the present study, FTIR analysis confirmed the co-presence of both alginate-specific signatures (asymmetric COO^−^ stretching at 1605 cm^−1^, indicative of Ca^2+^–alginate ionic crosslinking) and cellulose-specific signatures (C–O–C glycosidic bonds at 1020–1100 cm^−1^), while SEM micrographs showed cellulose microfibrils physically embedded within a continuous alginate matrix (Figure 2B–D), providing convergent spectroscopic and morphological evidence of a composite structure. Although the present study did not include an alginate-only control, as this would constitute a materials optimization investigation beyond the agronomic scope of this work, the well-documented reinforcing role of cellulose in polysaccharide-based hydrogels [32,33], combined with the FTIR and SEM evidence presented herein, supports the interpretation that the cellulose component contributes to the structural integrity and functional performance of the hydrogel in soil environments. To further elucidate the specific contribution of each component, we recommend that future studies include component-isolation experiments (alginate-only, cellulose-only, and composite formulations at various ratios) to quantify the specific contribution of each component to swelling behavior, mechanical stability, and agronomic performance.

### 2.9. Optimal Hydrogel Rate, Economics, and Practical Implications for Qatar

The 1% *w*/*w* hydrogel application rate demonstrated superior agronomic efficiency, with significant improvements in plant dry weight and water use efficiency compared to the 2% rate. Higher rates showed modest incremental benefits for most variables, suggesting diminishing returns and supporting 1% as the optimal dosage that balances water retention gains with material costs and aeration requirements in fine-rooted vegetable crops [12,16].

Date-palm waste valorization into biodegradable hydrogels provides economic and environmental advantages over synthetic superabsorbents. The date-palm residues used for cellulose extraction were collected from Qatar University campus at no material cost, eliminating the expensive virgin biomass acquisition required for synthetic superabsorbent polymers. Hydrogel synthesis required only inexpensive chemical materials (sodium alginate and calcium chloride) readily available at low costs. For the optimal 1% *w*/*w* application rate determined in this study, material costs per hectare are substantially lower than commercial synthetic superabsorbent products [13,38]. Furthermore, the improved soil water retention capacity directly reduces irrigation frequency, translating to measurable savings in water and labor costs, critical economic benefits in Qatar’s water-scarce environment. This preliminary study establishes a proof of concept for economic viability; a comprehensive cost–benefit analysis including production scaling, commercial feasibility, and field implementation will be completed upon project conclusion.

Demonstration of hydrogel efficacy in this controlled study establishes a proof of concept for improving soil hydro-physical properties and tomato productivity under Qatar’s arid conditions, aligning with the national food security strategy (70% self-sufficiency in vegetables by 2030) [30]. Importantly, the demonstrated performance of this crosslinked hydrogel system including rapid equilibrium swelling (5.659 g g^−1^ within 30 min), 42% increase in field capacity, and sustained moisture buffering under >40 °C conditions reflects the engineered polymer network architecture and cannot be replicated by uncomposted or composted date-palm biomass alone, which lacks the ionic crosslink structure (Ca^2+^–alginate junctions) and three-dimensional polymer network required for controlled, reversible water storage and release [4,9,13]. However, field-scale validation under variable irrigation regimes, soil types, and long-term degradation kinetics remains essential. Future research priorities include field trials with irrigation gradients (deficit, rainfed), mechanistic studies using rhizotron imaging or stable isotope labeling to quantify hydrogel–root water exchange pathways, biodegradation assessment over multiple seasons, multi-crop evaluation, economic and life-cycle analysis, and synthesis optimization for commercial scaling in Gulf protected agriculture.

## 3. Conclusions

This study successfully synthesized a biodegradable cellulose–alginate hydrogel from date-palm leaf biomass and demonstrated its efficacy for improving soil hydro-physical properties and tomato (*Solanum lycopersicum* L.) performance under controlled conditions. The hydrogel exhibited rapid hydration capacity (equilibrium SR = 5.659 g g^−1^, water uptake = 465.9% within 30 min) and was validated by FT-IR spectroscopy (O–H at 3340 cm^−1^, COO^−^ at 1605 cm^−1^, confirming Ca^2+^–alginate crosslinking), and SEM imaging showed a porous microstructure. Applied at the optimal 1% *w*/*w* rate, the hydrogel significantly enhanced soil water retention (porosity +12.4%, field capacity ~42%), maintained superior soil moisture under extreme evaporative demand (~2000 mm yr^−1^), and improved tomato fresh biomass by 38% and water use efficiency by 17% compared to the control.

While these results are promising under controlled conditions with a single tomato cultivar, several directions for future research should be highlighted. The composite hydrogel was evaluated as an integrated functional material; future studies incorporating component-isolation experiments (alginate-only, cellulose-only, and composite formulations at various ratios) would further elucidate the specific contribution of each polymeric component to the observed agronomic benefits. Additionally, field-scale validation under variable irrigation regimes and soil types, long-term degradation kinetics, and multi-crop evaluation remain essential before practical application. Future research should prioritize component-isolation experiments comparing alginate-only, cellulose-only, and hydrogel formulations to quantify the specific contribution of each polymeric component, field trials with irrigation gradients to quantify actual water savings, mechanistic studies using rhizotron imaging or stable isotope labeling to elucidate hydrogel–root–soil water exchange, biodegradation assessment over multiple seasons and soil types, and synthesis optimization for commercial scaling in Gulf-region protected agriculture. These investigations are critical for translating this proof of concept into viable agricultural technology.

## 4. Materials and Methods

The experiment was conducted during the summer season of 2024, from 12 June to 5 September, in a protected environment at the Research Complex of Qatar University, Doha, where daytime temperatures exceeded 40 °C and relative humidity ranged between 30 and 55 percent. This semi-controlled environment provided realistic arid-region conditions suitable for testing hydrogel–soil interactions.

### 4.1. Cellulose Extraction from Date-Palm Biomass

Fresh date-palm (*Phoenix dactylifera* L.) leaves collected from Qatar University campus were washed thoroughly to remove dust and impurities, cut into 4–6 cm pieces, and sun-dried, and ground into a fine powder. Cellulose was extracted following an alkali–bleaching method. A total of 80 g of leaf powder was mixed with 40 g NaOH in 600 mL deionized water, heated at 60 °C for 8 h, and washed until it reached a neutral pH. The material was subsequently bleached with sodium hypochlorite at 80 °C for 6 h and washed thoroughly. The extracted cellulose was dried at 70 °C to constant weight.

### 4.2. Synthesis of Cellulose–Alginate Hydrogel

The hydrogel synthesis employed a dispersion-in-matrix crosslinking approach. Sodium alginate (2.4 g) was dissolved in 150 mL of deionized water at 80 °C with continuous stirring for 1 h to form a viscous carrier solution. Extracted cellulose microfibrils (5 g) were then dispersed into the hot alginate matrix under high-shear mechanical stirring (800 rpm, 80 °C, 4 h). The elevated temperature and shear forces enabled thermal softening and partial fibrillation of cellulose microfibril bundles, facilitating uniform distribution throughout the alginate matrix while preserving the crystalline cellulose core structure. This dispersion step is critical because cellulose, being water-insoluble and hydrophobic, would otherwise aggregate and phase-separate. The cellulose–alginate dispersion was then filtered through a 200 µm mesh to remove any undispersed cellulose aggregates, and the filtrate was transferred into a calcium-mediated crosslinking bath (12 g CaCl_2_ dissolved in 100 mL deionized water, 12 h contact). During ionic gelation, Ca^2+^ ions formed electrostatic ‘egg-box’ junction zones by bridging guluronate and mannuronate blocks of adjacent alginate chains, creating a stable three-dimensional polymer network. Importantly, this crosslinking process physically entrapped and mechanically reinforced the dispersed cellulose microfibrils within the solidifying alginate gel.

### 4.3. Fourier Transform Infrared Spectroscopy (FT-IR)

FT-IR analysis was performed to confirm the chemical structure and functional groups of the date-palm-derived cellulose–alginate hydrogel. Dried hydrogel samples were finely ground and analyzed using a Nicolet 760 FTIR spectrometer (Nicolet Instrument Corporation, Madison, WI, USA) equipped with a potassium bromide (KBr) window. Approximately 5–10 mg of powdered sample was placed on the KBr plate, and spectra were recorded over 400–4000 cm^−1^ at room temperature. The FT-IR analysis followed the characterization procedure described for date-palm-derived cellulose by Al-Awa et al. [31].

### 4.4. Scanning Electron Microscopy (SEM)

The surface morphology and internal microstructure of the hydrogel were examined using a NovaNano SEM 450 (FEI, Hillsboro, OR, USA). Freeze-dried hydrogel samples were fractured to expose the internal cross-sections, mounted on aluminum stubs with conductive carbon tape, and sputter-coated with a thin Au/C layer to enhance conductivity. Imaging was performed at accelerating voltages between 200 V and 30 kV, and micrographs were captured at different magnifications to assess pore structure, surface roughness, and network formation. The procedure followed the SEM methodology previously applied for characterizing date-palm-derived cellulose [31].

### 4.5. Swelling Behavior and Water Uptake Measurements

The swelling behavior of the hydrogel was evaluated using a standard gravimetric method commonly applied to polysaccharide-based hydrogels [39,40]. Dried hydrogel samples were cut into uniform pieces, and their initial dry weight (*W_d_*) was recorded. The samples were then immersed in excess deionized water at 25 ± 1 °C. Swelling was monitored over defined time intervals from 0 to 180 min.

At each time point, the hydrogel samples were removed from the water, gently blotted with filter paper to eliminate surface-adhered moisture, and weighed to obtain the swollen mass (*W_t_*).

The swelling ratio (*SR*) at time *t* was calculated according to Equation (1):

(1) $$SR\left(t\right)=\frac{W_{t}-W_{d}}{W_{d}}$$

where *SR* represents the grams of water absorbed per gram of dry hydrogel (g g^−1^). Measurements were continued until equilibrium swelling was achieved.

Water uptake (%) was determined concurrently from the same gravimetric data and expresses the swelling behavior in percentage form relative to the dry hydrogel mass. It was calculated using Equation (2):

(2) $$\mathrm{Water}\;\mathrm{Uptake}\left(\%\right)=\frac{W_{t}-W_{d}}{W_{d}}\times 100=\left(SR-1\right)\times 100$$

This formulation highlights the direct mathematical relationship between the swelling ratio and water uptake, where water uptake (%) is the percentage expression of the swelling ratio. Both parameters were retained to ensure consistency with previously reported hydrogel characterization studies [39,41].

### 4.6. Plant Material and Seedling Establishment

Seeds of tomato plants (*Solanum lycopersicum* L., F1 hybrid) were obtained from the Technical Agricultural Company, Doha. Germination was carried out in nursery trays under controlled greenhouse conditions. After 30 days, uniform and healthy seedlings were transplanted into plastic pots (25 cm diameter × 20 cm height) containing a substrate mixture, with plants spaced 40 cm apart. Each plant was supported with a 1.5 m vertical stake and maintained through standard pruning and cultural practices.

### 4.7. Experimental Design and Growth Conditions

**Figure 8:**
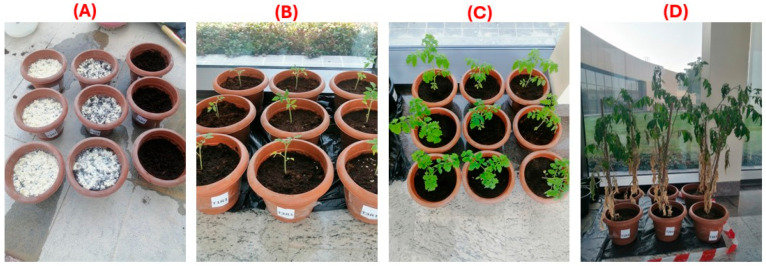
Effects of date-palm hydrogel on tomato growth. (**A**) Pots after hydrogel incorporation (0%, 1%, 2%). (**B**) Early seedling establishment. (**C**) Mid-season vegetative growth. (**D**) Flowering and maturity stage show enhanced plant performance in hydrogel treatments. Note: All nine experimental pots (3 treatments × 3 replicates) were maintained throughout the entire 86-day experiment. Panel D displays two of the three replicate rows; the third row was present but was not within the photographic frame at the time the image was taken.

A single-factor randomized complete design was employed to evaluate the effects of a date-palm-derived cellulose–alginate hydrogel on tomato growth. The hydrogel was synthesized using a fixed formulation and applied as an integrated soil amendment. Hydrogel concentration served as the sole treatment factor at three levels: 0% (T1, control), 1% (T2), and 2% (T3) *w*/*w* of soil dry weight. Each treatment was replicated three times, resulting in a total of nine experimental units. The growing medium consisted of organic manure, Qatari soil, and commercial potting soil mixed at a volumetric ratio of 1:2:1 (*v*/*v*/*v*). The Qatari soil was sourced from the Umm Bab region and pre-treated by the Ministry of Municipality for agricultural use. The substrate was homogenized and air-dried prior to pot filling. The physical and chemical characteristics of the soil are summarized in Table 3. Uniform tomato seedlings were transplanted into each pot and irrigated immediately after transplanting. Dry hydrogel at the designated application rate was thoroughly mixed with the soil and incorporated into 2 kg containers at a depth of 3 cm below the surface. Water was added until saturation to ensure complete hydration of both the hydrogel and soil mixture. Growth conditions, including germination, transplanting, irrigation, and fertilization practices, were selected based on established greenhouse protocols commonly applied under arid conditions. No additional optimization trials were performed within this study. Figure 8 illustrates the progression of plant growth under different treatment conditions, from initial soil preparation to the late stage.

### 4.8. Irrigation Scheduling

Hydrogel application rates (0%, 1%, and 2% *w*/*w* on a dry soil basis) were calculated prior to irrigation and thoroughly mixed with air-dried soil to ensure uniform distribution. Water was subsequently added to saturation to simultaneously hydrate the soil matrix and the incorporated hydrogel. Irrigation management was regulated based on volumetric soil moisture measurements, which were continuously monitored using a digital soil moisture sensor (Hunan Rika Electronic Tech Co., Ltd., Changsha, China), inserted 15 cm deep into the central root zone. Water was applied when soil moisture declined to approximately 60–65% of field capacity, typically every 3–4 days, resulting in approximately 24–25 irrigation events during the 86-day growing period. Each event supplied 350–450 mL of water, corresponding to a total seasonal irrigation depth of approximately 9.6–10.0 L (204 mm) per pot over the 86-day growing period. All treatments received identical irrigation quantities.

### 4.9. Soil Physical Measurements

Volumetric soil water content (%) was measured using digital soil moisture sensors (Hunan Rika Electronic Tech Co., Ltd., Changsha, China). The sensors were horizontally installed at the center of each pot to a depth of 15 cm, thereby capturing moisture dynamics within the active root zone. Soil moisture readings were taken both immediately before and after each irrigation event, and additional measurements were collected at regular intervals throughout the growing season. The field capacity (FC) and wilting point (WP) were determined following the procedure of Imakumbili [42], with modifications. Transparent plastic containers (7 cm diameter × 15 cm height) with drainage holes at the base were filled with experimental substrate. To determine FC, containers were saturated with water until free drainage occurred, covered to minimize evaporation, and allowed to drain in a protected corridor environment for 72 h. Soil samples were then collected from mid-depth, weighed fresh, and air-dried under ambient laboratory conditions until constant mass was achieved. Gravimetric water content at this equilibrium was taken as FC. To determine WP, tomato plants were grown in the pots until they reached wilting, defined as the inability to recover turgor after overnight rehydration. Soil samples were collected at this stage, weighed fresh, and airdried to constant mass. Gravimetric water content at wilting was taken as WP. Plant-available water content (PAWC) was calculated as ((FC − WP)/100) × BD, where BD is the bulk density of the soil. Soil bulk density (BD) was determined using the core method [43]. Total porosity (P) was calculated from bulk density and assumed particle density (2.65 g cm^−3^).

### 4.10. Plant Morphology and Physiology

Plant height, leaf number, and stem diameter were recorded at scheduled intervals. Stem diameter was measured 5 cm above the soil surface using a digital vernier caliper. Leaf area was determined using a portable laser leaf area meter (YMJ-B, TOP Instrument Co., Ltd., Hangzhou, China) following optical-scan principles [44]. Chlorophyll content and leaf nitrogen status were quantified using a Plant Nutrition Analyzer (Model TYS-3N/4N, TOP Instrument Co., Ltd., Hangzhou, China), a method consistent with chlorophyll–nitrogen correlations reported by Yang et al., [45]. Leaf relative water content (RWC) was calculated using the Smart and Bingham [46] method based on fresh, turgid, and dry leaf weights.

At the final stage, shoots (aboveground parts) and roots (belowground parts) were harvested separately. Plant material was washed with tap water to remove adhering soil particles and airdried under ambient laboratory conditions until constant mass was achieved. Fresh shoot and root weights were recorded using a digital balance, and dry weights were obtained after airdrying to constant mass. Root length was measured directly using a ruler.

### 4.11. Water Productivity

Water productivity was defined as the total dry biomass (g) produced per unit of irrigation water applied (kg). The total quantity of irrigation water required for each treatment (pot) was recorded at the end of the experiment and used to assess water use efficiency by dividing the plant’s total dry biomass by the total irrigation water applied per pot [10,34].

### 4.12. Statistical Analysis

All data were analyzed using one-way ANOVA, which is appropriate for the single-factor randomized design. Mean comparisons were performed using Tukey’s HSD at *p* ≤ 0.05. Statistical analysis was conducted using GraphPad Prism version 6.0.

## Figures and Tables

**Table 1:** Some soil hydro-physical parameters under three hydrogel application rates (treatments included hydrogel levels (T1, T2, T3 = 0, 1%, and 2% *w*/*w*)). Abbreviations: BD, soil bulk density; P, porosity (%); FC, field capacity (%); WP, wilting point (%); AW, available water (%); PAWC, plant-available water content (g/g). Data represents the mean of three replicates. Within each column, values marked with * are significantly different from T1 (control) at *p* < 0.05 according to Tukey’s multiple comparison test. Significance levels: ns = not significant; * = *p* ≤ 0.05; ** = *p* ≤ 0.01 for hydrogel treatments.

Treatment	BD (g/cm^3^)	P (%)	FC (%)	WP (%)	AW (%)	PAWC (g/g)
**T1**	1.38	47.92	21.17	8	13.13	0.18
**T2**	1.27 *	51.82 *	30.16 *	10.2 **	19.97 *	0.25 *
**T3**	1.22 *	54.10 *	30.93 **	11.1 **	19.83 *	0.24 ns
***p*-value** **(*p* < 0.05)**	0.0453	0.0469	0.0054	0.0028	0.0098	0.0176

**Table 2:** Growth parameters of tomato plants cultivated under three hydrogel application rates during the cropping season. Abbreviations: PFW, plant fresh weight; PDW, plant dry weight; RFW, root fresh weight; RDW, root dry weight; SFW, stem fresh weight; SDW, stem dry weight. Treatments included hydrogel levels (T1, T2, T3 = 0, 1%, and 2% *w*/*w*). Data represents the mean of three replicates. Within each column, values marked with * are significantly different from T1 (control) at *p* < 0.05 according to Tukey’s multiple comparison test. Significance levels: ns = not significant; * = *p* ≤ 0.05; ** = *p* ≤ 0.01; **** = *p* ≤ 0.0001 for hydrogel treatments.

Treatment	PFW (g)	PDW (g)	RFW (g)	RDW (g)	RL (cm)
**T1**	247.68	49.00	36	3.57 a	19.60
**T2**	341.70 **	68.68 *	42 *	4.48 **	25.10 *
**T3**	331.80 **	68.00 ns	47 ****	4.60 *	25.70 **
***p*** **-value (*p* < 0.05)**	0.0046	0.0403	0.0089	<0.0127	<0.0028

**Table 3:** The initial soil and date-palm hydrogel properties.

	Soil	Hydrogel
Sand (%)	40.53	-
Silt (%)	56.84	-
Clay (%)	2.63	-
Soil type	Sandy Silty	-
pH	7.73	7.5
EC (mS/sm^−1^)	2.91	1.0
TOM (%)	0.108	-
CEC (cmole/kg)	0.00423	-
CaCo_3_ (%)	42.56	0.006
P (mg/kg)	1.4243	1.47
K (mg/kg)	64.1383	2.03
Ca (mg/kg)	1259.578	2183.44
Mg (mg/kg)	152.6517	22.22
Na (mg/kg)	63.2937	689.13
Pb (mg/kg)	0.0145	0.42
Zn (mg/kg)	0.166	0.27

## Data Availability

The data presented in this study are available on request from the corresponding author.

## References

[1] Aldababseh A., Temimi M., Maghelal P., Branch O., Wulfmeyer V. (2018). Multi-Criteria Evaluation of Irrigated Agriculture Suitability to Achieve Food Security in an Arid Environment. Sustainability.

[2] Alhaj M., Mohammed S., Darwish M., Hassan A., Al-Ghamdi S.G. (2017). A review of Qatar’s water resources, consumption and virtual water trade. Desalination Water Treat..

[3] Al-Saidi M., Lahham N. (2019). Solar energy farming as a development innovation for vulnerable water basins. Dev. Pract..

[4] Agbna G.H., Shahab A., Zaidi S.J. (2025). Multifunctional hydrogel systems: Integrating nutrient delivery, soil enhancement, and climate resilience in modern agriculture. Desalination Water Treat..

[5] Agbna G.H.D., Zaidi S.J. (2025). Hydrogel Performance in Boosting Plant Resilience to Water Stress—A Review. Gels.

[6] Thombare N., Mishra S., Siddiqui M., Jha U., Singh D., Mahajan G.R. (2018). Design and development of guar gum based novel, superabsorbent and moisture retaining hydrogels for agricultural applications. Carbohydr. Polym..

[7] Ali K., Asad Z., Agbna G.H.D., Saud A., Khan A., Zaidi S.J. (2024). Progress and Innovations in Hydrogels for Sustainable Agriculture. Agronomy.

[8] Chang L., Xu L., Liu Y., Qiu D. (2021). Superabsorbent polymers used for agricultural water retention. Polym. Test..

[9] Guilherme M.R., Aouada F.A., Fajardo A.R., Martins A.F., Paulino A.T., Davi M.F., Rubira A.F., Muniz E.C. (2015). Superabsorbent hydrogels based on polysaccharides for application in agriculture as soil conditioner and nutrient carrier: A review. Eur. Polym. J..

[10] Albalasmeh A.A., Mohawesh O., Gharaibeh M.A., Alghamdi A.G., Alajlouni M.A., Alqudah A.M. (2022). Effect of hydrogel on corn growth, water use efficiency, and soil properties in a semi-arid region. J. Saudi Soc. Agric. Sci..

[11] Muhammad N., Kader M.A., Al-Solaimani S.G., El-Wahed M.H.A., Abohassan R.A., Charles M.E. (2025). A review of impacts of hydrogels on soil water conservation in dryland agriculture. Farming Syst..

[12] Saha A., Sekharan S., Manna U. (2020). Superabsorbent hydrogel (SAH) as a soil amendment for drought management: A review. Soil Tillage Res..

[13] Oladosu Y., Rafii M.Y., Arolu F., Chukwu S.C., Salisu M.A., Fagbohun I.K., Muftaudeen T.K., Swaray S., Haliru B.S. (2022). Superabsorbent Polymer Hydrogels for Sustainable Agriculture: A Review. Horticulturae.

[14] Abdelghafar R., Abdelfattah A., Mostafa H. (2024). Effect of super absorbent hydrogel on hydro-physical properties of soil under deficit irrigation. Sci. Rep..

[15] Islam M.R., Hu Y., Mao S., Mao J., Eneji A.E., Xue X. (2011). Effectiveness of a water-saving super-absorbent polymer in soil water conservation for corn (*Zea mays* L.) based on eco-physiological parameters. J. Sci. Food Agric..

[16] El Idrissi A., Dardari O., Metomo F.N.N.N., Essamlali Y., Akil A., Amadine O., Aboulhrouz S., Zahouily M. (2023). Effect of sodium alginate-based superabsorbent hydrogel on tomato growth under different water deficit conditions. Int. J. Biol. Macromol..

[17] Ullah F., Othman M.B.H., Javed F., Ahmad Z., Akil H.M. (2015). Classification, processing and application of hydrogels: A review. Mater. Sci. Eng. C.

[18] Maitra J., Shukla V.K. (2014). Cross-linking in hydrogels—A review. Am. J. Polym. Sci.

[19] Alrumman S.A. (2016). Enzymatic saccharification and fermentation of cellulosic date palm wastes to glucose and lactic acid. Braz. J. Microbiol..

[20] Alkoaik F., Al-Faraj A., Al-Helal I., Fulleros R., Ibrahim M., Abdel-Ghany A.M. (2019). Toward Sustainability in Rural Areas: Composting Palm Tree Residues in Rotating Bioreactors. Sustainability.

[21] Shaikh H.M., Anis A., Poulose A.M., Al-Zahrani S.M., Madhar N.A., Alhamidi A., Alam M.A. (2021). Isolation and Characterization of Alpha and Nanocrystalline Cellulose from Date Palm (*Phoenix dactylifera* L.) Trunk Mesh. Polymers.

[22] Alsubaie F.S., Srdar M., Fayraa O., Alsulami F.M., Omran F., Alamry K.A. (2025). Development of Eco-Friendly Date Palm Biomass-Based Hydrogels for Enhanced Water Retention in Soil. Gels.

[23] Abid W., Ammar E., Ramadan M.F., Farag M.A. (2022). Date Palm (*Phoenix dactylifera* L.) Wastes Valorization: A Circular Economy Approach. Mediterranean Fruits Bio-Wastes.

[24] Faiad A., Alsmari M., Ahmed M.M.Z., Bouazizi M.L., Alzahrani B., Alrobei H. (2022). Date Palm Tree Waste Recycling: Treatment and Processing for Potential Engineering Applications. Sustainability.

[25] Alonso-Cuevas C.F., Ramírez-Guzmán N., Serna-Cock L., Guancha-Chalapud M., Aguirre-Joya J.A., Aguillón-Gutiérrez D.R., Claudio-Rizo A., Torres-León C. (2025). From Agro-Industrial Waste to Natural Hydrogels: A Sustainable Alternative to Reduce Water Use in Agriculture. Gels.

[26] Agbna G.H., Talha Z., Siddig N., Osman M.M.M. (2017). Biochar amendments improves tomato growth, yield and irrigation water use efficiency under poor silt loam soil. Int. J. Eng. Scient. Res..

[27] Agbna G.H., Dongli S., Zhipeng L., Elshaikh N.A., Guangcheng S., Timm L.C. (2017). Effects of deficit irrigation and biochar addition on the growth, yield, and quality of tomato. Sci. Hortic..

[28] Nangare D.D., Singh Y., Kumar P.S., Minhas P.S. (2016). Growth, fruit yield and quality of tomato (*Lycopersicon esculentum* Mill.) as affected by deficit irrigation regulated on phenological basis. Agric. Water Manag..

[29] Patanè C., Tringali S., Sortino O. (2011). Effects of deficit irrigation on biomass, yield, water productivity and fruit quality of processing tomato under semi-arid Mediterranean climate conditions. Sci. Hortic..

[30] Amhamed A., Genidi N., Abotaleb A., Sodiq A., Abdullatif Y., Hushari M., Al-Kuwari M. (2023). Food security strategy to enhance food self-sufficiency and overcome international food supply chain crisis: The state of Qatar as a case study. Green Technol. Resil. Sustain..

[31] Al-Awa Z.F.A., Sangor F.I.M.S., Babili S.B., Saud A., Saleem H., Zaidi S.J. (2023). Effect of Leaf Powdering Technique on the Characteristics of Date Palm-Derived Cellulose. ACS Omega.

[32] Omidian H., Akhzarmehr A., Chowdhury S.D. (2024). Advancements in Cellulose-Based Superabsorbent Hydrogels: Sustainable Solutions across Industries. Gels.

[33] Shaheen W., Iqbal M.M., Qudrat L. (2025). Development of cellulose-based superabsorbent polymers: A review. Cellulose.

[34] Hatfield J.L., Dold C. (2019). Water-Use Efficiency: Advances and Challenges in a Changing Climate. Front. Plant Sci..

[35] Krasnopeeva E.L., Panova G.G., Yakimansky A.V. (2022). Agricultural Applications of Superabsorbent Polymer Hydrogels. Int. J. Mol. Sci..

[36] Rani N., Kumar N. (2025). Hydrogel Polymer: A Water Conservation Practice for Drought Prone Agriculture. Int. J. Environ. Sci..

[37] Ungureanu E., Mikhailidi A., Tofanica B.-M., Fortună M.E., Rotaru R., Ungureanu O.C., Samuil C., Popa V.I. (2025). Sustainable Gels from Polysaccharides in Agriculture. Polysaccharides.

[38] Koushal S., Vishnoi M., Pj R., Vishnoi V., Premkumar, Singh K. (2025). Hydrogels as a key solution for sustainable agriculture: Exploring water retention and soil improvement. Int. J. Res. Agron..

[39] Chang C., He M., Zhou J., Zhang L. (2011). Swelling Behaviors of pH- and Salt-Responsive Cellulose-Based Hydrogels. Macromolecules.

[40] Chirani N., Yahia L.H., Gritsch L., Motta F.L., Chirani S., Farè S. (2015). History and applications of hydrogels. J. Biomed. Sci..

[41] El Idrissi A., El Gharrak A., Achagri G., Essamlali Y., Amadine O., Akil A., Sair S., Zahouily M. (2022). Synthesis of urea-containing sodium alginate-g-poly(acrylic acid-co-acrylamide) superabsorbent-fertilizer hydrogel reinforced with carboxylated cellulose nanocrystals for efficient water and nitrogen utilization. J. Environ. Chem. Eng..

[42] Imakumbili M.L.E. (2019). Making Water Stress Treatments in Pot Experiments: An Illustrated Step-by-Step Guide.

[43] Blake G.R., Hartge K.H. (1986). Bulk Density. Methods of Soil Analysis: Part 1 Physical and Mineralogical Methods.

[44] Wang T., Han J., Fang H., Khan A.A., Tang L., Zhang M., Shi F. (2021). The enhanced functional traits contribute to the successful invasion of Amaranthus palmeri in salinity environments: A comparison with its congeners. Biologia.

[45] Yang J., Shi S., Gong W., Du L., Ma Y., Zhu B., Song S. (2015). Application of fluorescence spectrum to precisely inverse paddy rice nitrogen content. Plant Soil Environ..

[46] Smart R.E., Bingham G.E. (1974). Rapid Estimates of Relative Water Content. Plant Physiol..

